# Separation of Active Compounds from Food by-Product (Cocoa Shell) Using Subcritical Water Extraction

**DOI:** 10.3390/molecules23061408

**Published:** 2018-06-11

**Authors:** Stela Jokić, Tanja Gagić, Željko Knez, Drago Šubarić, Mojca Škerget

**Affiliations:** 1 Faculty of Food Technology Osijek, Josip Juraj Strossmayer University of Osijek, Franje Kuhača 20, 31000 Osijek, Croatia; drago.subaric@ptfos.hr; 2 Faculty of Chemistry and Chemical Engineering, University of Maribor, Smetanova 17, 2000 Maribor, Slovenia; tanja.gagic@um.si (T.G.); zeljko.knez@um.si (Ž.K); mojca.skerget@um.si (M.Š.); 3 Faculty of Medicine, University of Maribor, Taborska ulica 8, 2000 Maribor, Slovenia

**Keywords:** cocoa shell, subcritical water extraction, active compounds

## Abstract

Large amounts of residues are produced in the food industries. The waste shells from cocoa processing are usually burnt for fuel or used as a mulch in gardens to add nutrients to soil and to suppress weeds. The objectives of this work were: (a) to separate valuable compounds from cocoa shell by applying sustainable green separation process—subcritical water extraction (SWE); (b) identification and quantification of active compounds, sugars and sugar degradation products in obtained extracts using HPLC; (c) characterization of the antioxidant activity of extracts; (d) optimization of separation process using response surface methodology (RSM). Depending on applied extraction conditions, different concentration of theobromine, caffeine, theophylline, epicatechin, catechin, chlorogenic acid and gallic acid were determined in the extracts obtained by subcritical water. Furthermore, mannose, glucose, xylose, arabinose, rhamnose and fucose were detected as well as their important degradation products such as 5-hydroxymethylfurfural (5-HMF), furfural, levulinic acid, lactic acid and formic acid.

## 1. Introduction

Cocoa shells are usually considered as “waste” and mainly used in the preparation of animal feed, fertilizer and fuel. So far, the main purpose (application) of cocoa shells in the food industry was as a source of fiber [1,2,3]. In the last few years, this raw material has been recognized as a valuable by-product of chocolate production, readily available worldwide in large quantities [3].

Cocoa shells contain a small proportion of fat with a very interesting fatty acid profile with a predominance of palmitic (22.27%) and oleic (28.16%) acids, similar to that of cocoa butter [3,4]. Aside from these dominant fatty acids, cocoa shells also contain capric (16.89%), stearic (12.05%), and linoleic (7.49%) acids and few others [4]. A relatively small proportion of fat and a small proportion of soluble sugars [5,6] are other advantages of this potential raw material, what makes it a useful ingredient for energy-reduced functional foods [7]. 

It was also proved that cocoa shell is rich in dietary fibre. Insoluble fibre mainly contains glucose and uronic acid. Mannose, galactose, xylose, arabinose, rhamnose and fucose are also present, but in lower amounts [1]. 

Cocoa shell is rich also in protein, theobromine, theophylline, caffeine [3,8,9], water soluble pectins [10] and ash [3]. Theobromine, which is considered toxic, has an extensive pharmacological function, is also a powerful diuretic, promotes diuresis and stimulates circulation failure, and consequently helps to remove harmful substances by urinary tract [8]. Caffeine, theophylline and theobromine, although having similar effect on the body such as the stimulation of the nervous system, heart and skeletal muscle and also muscle relaxation, differ in their intensity of action [3].

Recently, Okiyama et al. [3] have reviewed the content of some other valuable bioactive components in cocoa shell such as phenolic compounds, popular due to their antioxidant activity that can defend cells from oxidative damage. The amount of polyphenols in cocoa shell extracts as well as its biological activity, depends mostly on the nature of the extraction solvent used and on the presence of other bioactive components [11]. Some other important active compounds of cocoa shells are tannins, anthocyanins and proanthocyanidins, also known for their strong antioxidant activity [10]. 

For the extraction of bioactive compounds such as phenolic compounds water in the subcritical state can be used. Water represents safe, cheap and environmentally friendly solvent. Thus, water as an extraction medium can produce clean and safe extracts without environmental pollution and also decreases the costs of extraction processes. On the other hand, water is a polar compound at ambient conditions, therefore the extraction of less polar and non-polar compounds is very difficult. This drawback can be overcome if water is in a subcritical state [12]. Increasing the temperature above its normal boiling point (100 °C) to its critical point (374 °C) and under sufficient pressure, water retains its liquid form, but becomes less polar and more similar to organic solvents [13]. Subcritical water can extract different classes of compounds: lower temperature conditions are suitable for the extraction of more polar constituents, while higher temperatures make subcritical water as an extraction medium for non-polar ones [14]. Furthermore, conventional extraction techniques require longer extraction times compared to subcritical water extraction. Finally, another advantage of subcritical water extraction is that obtained extracts can be lyophilized and used in subsequent processes [12]. The use of these new green processes opens the possibility to achieve better product qualities and/or even to allow for the production of completely new products for use in the food, beverage, cosmetic and pharmaceutical industries as natural ingredients [12].

The objectives of this work were: (a) to separate valuable compounds from cocoa shell by applying sustainable separation process—subcritical water extraction (SWE); (b) identification and quantification of active compounds in obtained extracts using HPLC; (c) characterization of antioxidant activity of extracts; (d) optimization of the SWE process using response surface methodology (RSM).

## 2. Results and Discussion

This is the first study to explore the SWE of cocoa shell so comparisons of our results to literature data on the same extraction method and material are limited, especially with regard to details about the bioactive compound and sugar content of the resulting extract. As already mentioned, cocoa shell is rich in methylxanthines (theobromine, theophylline and caffeine) and contains some phenolic compounds, and a suitable extraction of these constituents is the first step in the utilization of such bioactive compounds. The methylxanthine and phenolic compounds identified in the cocoa shell extracts in this study obtained by BBD are presented in Table 1, where results are expressed in weight percentage (% *w*/*w*).

### 2.1. Phenolic Compounds and Antioxidant Activity of Cocoa Shell

The strong antioxidant activity of phenolic compounds may protect cell components from oxidative damage, consequently, it can also limit the risk of several degenerative diseases associated with oxidative stress [15]. One of the factors that influence the extraction of phenolic compounds from plant material is the chemical nature of the compounds, while the chosen extraction method and the applied extraction parameters have a particularly significant effect [16].

According to Nazaruddin et al. [17], phenolic compounds are stored in cocoa seed cotyledons; however, some of these compounds become lost due to diffusion from cotyledons during the fermentation. As a result of this process, many of these bioactive compounds are concentrated in the cocoa shells. Azizah et al. [18] showed a strong antioxidant activity of phenolic compounds found in cocoa shells, when compared to synthetic antioxidants.

The results of our study show that TP content in obtained extracts of cocoa shell can differ significantly according to the applied extraction conditions of SWE from 27.26 (SWE conditions: 120 °C, 75 min, 20 mL/g) to 130.33 mg GAE/g (SWE conditions: 220 °C, 75 min, 20 mL/g) and antioxidant activity ranged from 19.20% DPPH scavenging (SWE conditions: 120 °C, 75 min, 20 mL/g) to 91.69% DPPH scavenging (SWE conditions: 220 °C, 75 min, 20 mL/g). From obtained results positive and very good correlation between TP content and antioxidant activity is obvious. Furthermore, the predominant effect of temperature on the SWE process is observed which is proven in the statistical results given in Table 2. 

It has been already mentioned that there are no reports about phenolic content in subcritical water extracts of cocoa shell, as reports are mainly on classical extracts obtained by organic solvents and few of them on extracts obtained by supercritical CO_2_. The latest published studies are related to pulsed electric field assisted extraction [19] and integrated green-based processes using supercritical CO_2_ and pressurized ethanol [20] to recover bioactive compounds from cocoa shells. For example, Ismail and Lea [21] reported that TP of cocoa shell was 112.90 mg/g (expressed as gallic acids equivalents in mg per g of extract obtain with 70% ethanol solution) and the free radical scavenging activity was 95.9%. We already mentioned [17] that high phenolic content in cocoa shell could be due to the migration of some phenolics from cocoa cotyledons to its shell during fermentation because cocoa cotyledons are rich in phenolic compounds such as catechin, epicatechin and procyanidins [22,23]. In recent studies by Hernández-Hernández et al. [24] the cocoa shell samples obtained after fermentation have important concentrations of bioactive compounds (mainly theobromine, epicatechin and catechin, obtained by organic solvent extraction) which, together with its high fiber content and antibacterial activity, could make it an interesting novel ingredient for the food industry. Martinez et al. [25] also considered that cocoa shell by-product may be a potential source of natural compounds with significant antioxidant activity. TP ranged from 80.17 to 144.83 mg/100 g of samples, while methanol:acetone extracts provides higher antioxidant activity values compared to ethanol extracts. Arlorio et al. [26] investigated the antioxidant activity and phenolics of the cocoa shell extracts obtained by supercritical CO_2_. The results showed that extracts rich in phenolic compounds are quite interesting and can be applied as colorants and as sources of bioactive compounds with functional properties.

Mazzutti et al. [20] investigated the TP content and antioxidant activity of cocoa shell extracts obtained by different extraction techniques (supercritical fluid extraction, pressurized ethanol extraction and integrated green-based process) and showed that an integrated green-based process gave the highest TP (from 35 to 51 mg GAE/g depending on applied extraction conditions) and the highest antioxidant activity while supercritical CO_2_ extracts, pressurized ethanol extraction and Soxhlet gave low content of TP and lower antioxidant activity. Barbosa-Pereira et al. [19] were focused on the application of pulsed electric fields as an innovative pre-treatment technique to improve the recovery of polyphenols from cocoa shell and showed that approximately 20% higher recovery yields of polyphenols and methylxanthines compared to conventional extraction were obtained. TP in cocoa shell extracts varied from 24.93 to 32.30 mg GAE/g of dry weight according to central composite design.

Only a few studies have focused on the individual phenolic content in cocoa shell. The major phenolic compounds identified in cocoa are flavanols (catechins, epicatechins, procyanidins) [3] present mainly in cotyledons and part of them can migrate to cocoa shell, generating a material enriched in these compounds. 

Hernández-Hernández et al. [24] published that epicatechin was the most abundant flavonoid in cocoa shell (6.93–17.70 mg/g) followed by catechin (1.02–6.16 mg/g). Different concentrations of these compounds were obtained according to the extraction solvent applied.

The major individual cocoa shell phenolic compound identified in our study in all 17 SWE runs was epicatechin, while catechin and chlorogenic acid were also detected in some extracts, but in lower concentration and only traces of gallic acid were observed. From the obtained results it can be noticed that chlorogenic acid and catechin were detected only at temperatures higher than 220 °C. Chlorogenic acid is known to have numerous medicinal properties, including strong antioxidant activity in addition to hepatoprotective, hypoglycemic and antiviral functions [27]. Catechin and epicatechin, as flavonoid compounds, represent extremely important cocoa shell products. Beside their high antioxidant activity, they have ability to decrease oxidative stress [28] and improve cardiovascular function [29].

### 2.2. Methylxanthines of Cocoa Shell

Methylxanthines are known for their psychoactive effects and because of that are interesting compounds in some products. Theobromine is considered a toxic compound, but it is reported that it also possesses many pharmacological activities such as anticancer, diuretic, cardio-stimulant, hypocholesterolemic, smooth-muscle relaxant, anti-asthma and coronary vasodilator properties [30]. When cocoa beans are processed, the theobromine content changes mainly during the fermentation stage. During this stage, methylxanthines migrate from the bean into the shell, causing a decrease in cocoa bean theobromine content of about 25% [31].

The data in Table 1 indicate that methylxanthines (theobromine and caffeine) were the most abundant compounds. Theophylline was detected only at extraction temperatures higher then 220 °C. The concentration of theobromine ranged from 1.63% to 4.77% (*w*/*w*), while the concentration of caffeine ranged from 0.04% to 0.29% (*w*/*w*). 

From Table 1 it is obvious that the theobromine concentration in the extract increases from 120 °C to 170 °C and after that it decreases. The possible explanation is that the detected caffeine is converted into theobromine and at higher reaction temperatures they both degrade into minor compounds.

Theobromine is an important compound in the cocoa shell, whose content is around 12.9 mg/g of dry weight for a mixture of cocoa shells from different geographic regions [26]. Barbosa-Pereira et al. [19] published that level of theobromine in cocoa shell changed with the origin of cocoa and varied between 4.64 and 10.92 mg/g, while caffeine content ranged between 1.59 and 4.21 mg/g. Carrillo et al. [32] found caffeine levels from different cultivars in the range 0.73–1.73 mg/g which confirms the fact that the caffeine level depends on the origin of cocoa and cultivars.

**Scheme 1:**
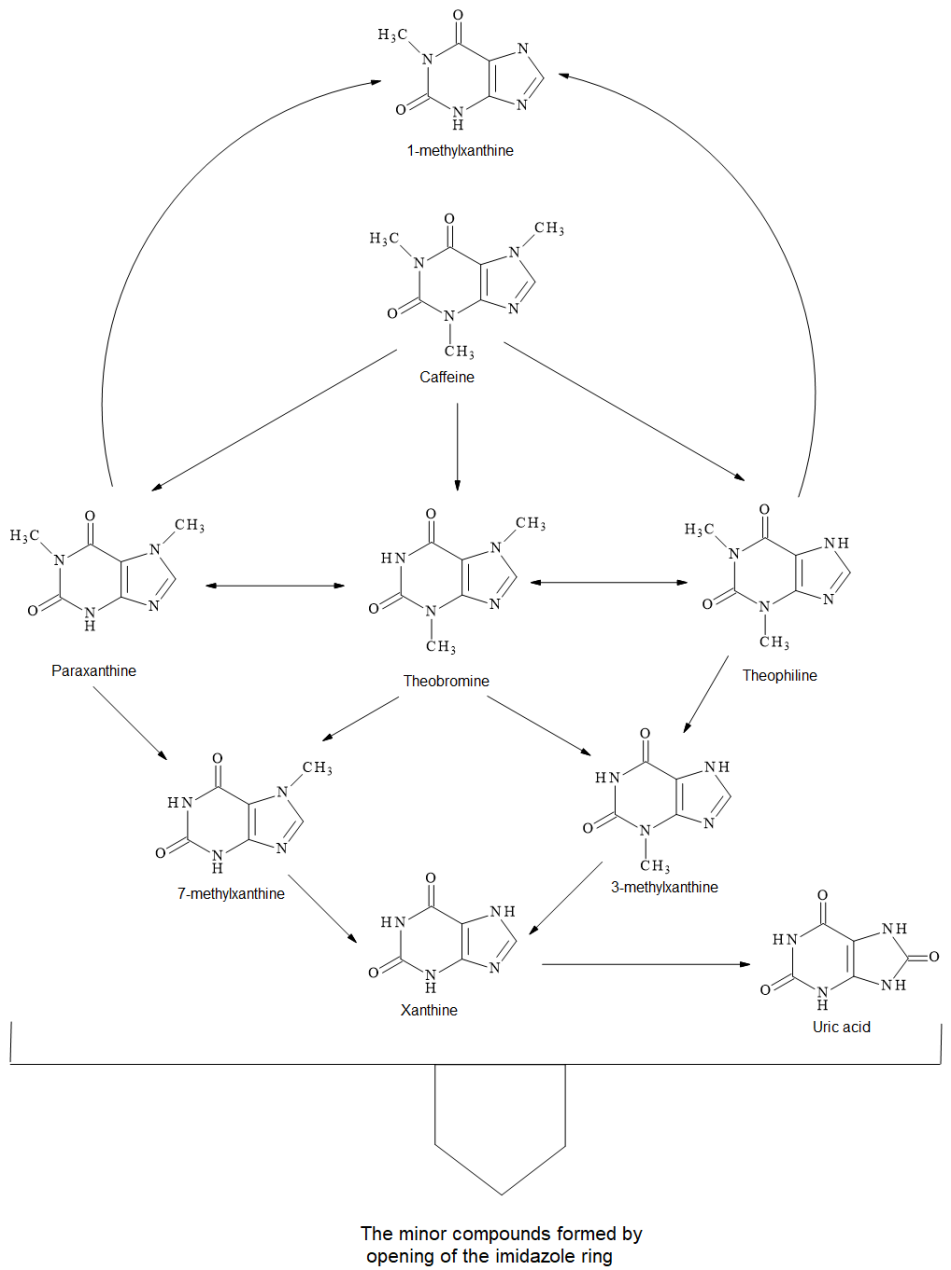
Proposed degradation mechanism of methylxanthines within hydrothermal degradation of cocoa shell.

A possible degradation mechanism of methylxanthines is shown in Scheme 1 based on our results and the literature [33,34]. Caffeine can degrade into theobromine, theophylline and paraxanthine, and also into 1,3,7-methyluric acid. Simultaneously, theobromine can isomerize into theophylline and paraxanthine. Theobromine can possibly degrade into 3-methylxanthine and 7-methylxanthine, which could also be formed from paraxanthine, while 3-methylxanthine can be formed from theophylline. 1-Methylxanthine could be a product of both paraxanthine and theophylline. All these methylxanthines can form uric acids by hydroxylation. Furthermore, the opening of the imidazole rings of methylxanthines can also form other minor compounds. 

### 2.3. Response Surface Analysis and Optimization

A Box-Behnken design (BBD) was used to optimize the most important operating variables of the SWE extraction (temperature, time and solvent-solid ratio) in order to achieve the higher amount of targeted bioactive compounds (methylxanthines and phenolics). Different SWE extraction parameters also influenced the abundance of the compounds in the obtained extracts. The coefficients and the corresponding *p*-values for each investigated responses are given in Table 2. The regression coefficients for linear, quadratic were determined by using multiple linear regression. The degree of statistical significance of each factor is represented with *p*-value. From Table 1 it can be noticed that only four of the responses (theobromine, caffeine, TP and antioxidant activity, respectively) are detected in all 17 runs so they were used in further calculations. The linear term of temperature exhibited the most statistically significant influence on all investigated responses (*p* < 0.05), while linear term of time did not show any significant influence on investigated responses. Linear term of solvent-solid ratio showed only significant influence on TP (*p* = 0.007) and antioxidant activity (*p* = 0.035). Interaction between input variables showed statistically significant influence only on TP and antioxidant activity (*X*_1_*X*_2_) while on theobromine and caffeine content were non-significant (*p* ≥ 0.05).

The statistical significance of regression equations for each selected response (theobromine, caffeine, total phenols and DPPH value) was evaluated by analysis of variance (ANOVA) and given in Table 3. The regression models for all investigated responses were highly significant according to the *p*-value (from 0.002 to 0.013) with satisfactory coefficients of determination (*R*^2^) ranging from 0.89 to 0.93. The non-significant lack-of-fit (*p* > 0.05) for each response highlighting that the second order polynomial model is adequate and could be used for precision of experimental values.

**Figure 1:**
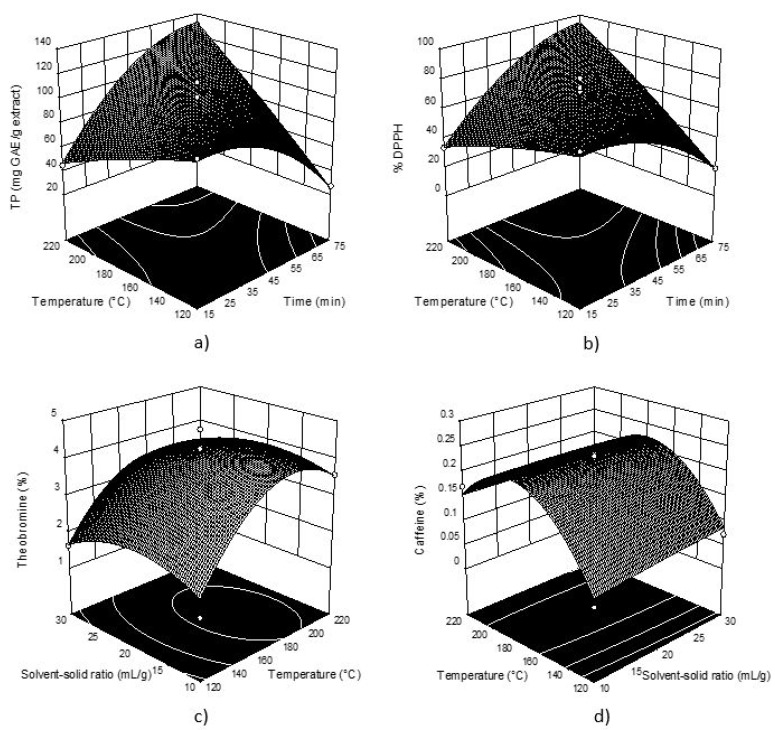
Three-dimensional plots for obtained TP (**a**) and DPPH (**b**) in cocoa shell extracts as a function of extraction temperature and time; Three-dimensional plots for for theobromine (**c**) and caffeine (**d**) as a function of extraction temperature and solid-solvent ratio respectively.

Considering the obtained values, these data were used for creating 3D graphs of the selected response surface (Figure 1). The plots were obtained depicting two variables (the most significant) within the experimental range, while the third variable was fixed constant at its respective center value of the testing range.

TP and DPPH show very similar response plot shapes (Figure 1), which was expected according to well-known positive correlation between TP and antioxidant activity and the obtained response plots indicate the same influence of process parameters on both the investigated responses (interactions between temperature and time had the most statistically significant influence). It can be seen that the TP content and DPPH decreased with the increase of extraction temperature, while applying longer extraction time TP content and % DPPH decreased. 

Figure 1 also shows the most abundant methylxanthines in cocoa shell extracts, primarily theobromine, where it can be seen that temperature had a significant influence on the obtained content. For theobromine and caffeine an increase of temperature higher than 170 °C shows a decrease in their content, while solvent-solid ratio had no significant influence. 

The final goal of RSM is the process optimization where the developed model can be used for simulation of the process for those components/responses that are present in all the obtained SWE extracts. It is important that the solvent-solid ratio is as small as possible but at the same time big enough to provide the highest possible extraction yield [35]. The results showed that temperature represents an extremely important parameter due to the fact that at different temperatures different components were formed. Because of that it is important to optimize the extraction procedure. By applying the desirability function method, the optimal conditions were calculated to be temperature of 170 °C, time of 75 min and solvent-solid ratio 20 mL/g. Under these optimal conditions, the predicted theobromine content was 3.88%, caffeine 0.22%, TP 75.25 mg GAE/g extract and DPPH scavenging activity of extract was 56.11%. These predicted data were experimentally confirmed with a deviation of ±5%. 

In many works as published in reviews of Plaza and Turner [36] and Okiyama et al. [3], a higher antioxidant capacity has been observed in the extracts obtained at temperatures over 175 °C and at longer extraction times, compared to the extracts obtained at lower temperature and shorter extraction times. In many of these studies, only the TP and the total antioxidant capacity were measured, with drawbacks of quantification of individual bioactive compounds.

### 2.4. Sugar Content of Cocoa Shell

The composition of cocoa shell also includes polysaccharides. It is proved in some patents [37,38] that cocoa shell predominantly contains cellulose units with lower amounts of pectin and hemicellulose. Redgwell et al. [6] studied the polysaccharide composition of cocoa shell and by different methods they identified glucose, galactose, mannose, xylose, arabinose, fucose, rhamnose and uric acid. In the present work similar sugars were found in cocoa shell extract, but also sugar derivatives, such as 5-HMF and furfural, and organic acids (formic, levulinic and lactic acid). The concentrations of sugars and their degradation products are presented in Table 4.

**Scheme 2:**
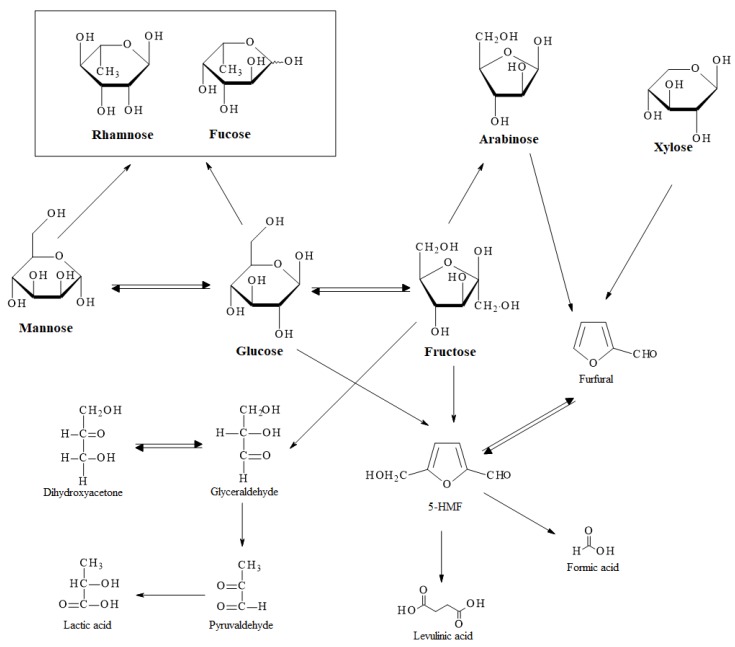
Proposed mechanism of sugar degradation within hydrothermal degradation of cocoa shell.

Scheme 2 presents a possible mechanism for the sugar degradation which takes place when cocoa shell is hydrothermally treated, proposed based on the literature [39,40] and our results. At a temperature of 120 °C the only sugar was mannose, which isomerized into glucose at a temperature of 170 °C. Glucose probably isomerized into fructose, which further lost a −CH_2_O group to form arabinose. Rhamnose and fucose were probably products of mannose and glucose. It can be noticed that the sugar concentrations increased with an increase of reaction time and temperature and decreased with an increase of concentration of initial material. 5-HMF can be obtained directly from glucose or through fructose, which further formed furfural. Furfural also represents a potential product of arabinose and xylose. At a temperature of 120 °C, 5-HMF and furfural were not detected in the extract, while at 170 °C they were present in trace amounts. At a temperature of 220 °C and reaction time of 15 min, 5-HMF and furfural reached the maximal concentrations and as the reaction time was further increased their concentration decreased again. At a temperature higher than 220 °C and longer reaction times, the main products were organic acids. The detected organic acids were levulinic, lactic and formic acids. Levulinic and formic acids were obtained from 5-HMF while lactic acid was probably produced through glyceraldehyde and pyruvaldehyde which were the products of fructose.

## 3. Materials and Methods

### 3.1. Material

Cocoa shells, a by-product of the Kandit chocolate factory (Osijek, Croatia) were obtained in 2017. Before the extraction, the plant material was ground using a standard laboratory mill. DPPH and ethyl acetate were purchased from Sigma-Aldrich Chemie (Steiheim, Germany). All other solvents were of analytical grade and purchased from J.T. Baker (J.T. Baker, Phillipsburg, NJ, USA). Folin-Ciocalteau’s phenol reagent was purchased from Sigma-Aldrich Chemie (Steiheim, Germany). Caffeine (purity 99%), theobromine (purity 98.5%), catechin (purity 99%), epicatechin (purity 97%) gallic acid (purity 99%) and chlorogenic acid (purity 98%) were purchased from Sigma-Aldrich. Theophylline (purity 99%), furfural (purity 99%) and 5-HMF (purity 98%) were purchased from Acros Organics (Geel, Belgium). Sugars (glucose, rhamnose, arabinose, mannose, xylose and fucose) and carboxylic acids (levulinic acid, lactic acid and formic acid) with high-purity (>99%) for HPLC analysis were obtained from Sigma-Aldrich.

### 3.2. Subcritical Water Extraction (SWE)

SWE of cocoa shell was carried out in a 75 mL batch reactor (series 4740 Stainless Steel, Parr Instruments, Moline, IL, USA) designed for a maximum operating temperature of 540 °C and pressure of 580 bar. The extractions were performed at temperatures of 120, 170 and 220 °C and reaction times of 15, 45 and 75 min. Different concentrations of initial material were used (1/10 g/mL, 1/20 g/mL, 1/30 g/mL). The influence of temperature, reaction time and concentration of cocoa shell suspension on product concentrations were observed. Cocoa shell—water mixture was poured into the reactor. The reactor was heated with an electrical wire. The mixture was stirred at 600 rpm. The CO_2_ was used to control pressure and remove present oxygen, to avoid oxidation reactions. When the extraction finished, the reactor was rapidly cooled in an ice bath. The reactor content was filtrated by vacuum filtration and the obtained products were water-soluble phase (extract) and solid residue (remaining cocoa shell or char). The solid residue was washed with water to collect the rest of the soluble components. Thus obtained extracts were analyzed by HPLC and UV spectrophotometer. The yield of extract was calculated by Equation (1):

(1) $$Y\;\left(\%\right)=\frac{m_{extract}}{m_{initial\;material}}\times 100$$

### 3.3. Experimental Design

A Box–Behnken design (BBD), explained in detail by Bas and Boyaci [41], was used for determination of the optimal process conditions for the SWE extraction of cocoa shells. The extraction temperature (*X*_1_), time (*X*_2_) and solvent-solid ratio (*X*_3_) were independent variables studied to optimize the extraction process in terms of getting higher amounts desired of active components in the extracts. Experimental data were fitted with second order response surface model with the following form (Equation (2)):

(2) $$Y=\beta_{0}+\overset{k}{\underset{i=1}{\sum }}\beta_{i}X_{i}+\overset{k}{\underset{i=1}{\sum }}\beta_{ii}X_{i}^{2}+\overset{k-1}{\underset{\begin{array}{c} i=1\\ i<j \end{array}}{\sum }}\overset{k}{\underset{j=2}{\sum }}\beta_{ij}X_{i}X_{j}$$

where *Y* are investigated responses (methylxanthines, phenolic compounds, total phenols and antioxidant activity, in SWE extracts, respectively), *β*_0_, *β*_i_, *β*_ii_, *β*_ij_ are constant coefficients of intercept, linear, quadratic, and interaction terms, respectively; *X*_i_ and *X*_j_ are coded independent variables. 

### 3.4. HPLC Method for Determination of Phenolic Compounds and Methylxanthines

An Agilent 1100 Series HPLC system (Agilent Technologies, Waldbronn, Germany), equipped with binary pump, an autosampler, a column heater and variable wavelength detector (VWD), was used for analysis of phenolic compounds and methylxanthines from extracts. The C18 column (4.0 × 250 mm, 5 μm particle size) was used. Two mobile phases were used: water- formic acid (99.5:0.5) as solvent A and acetonitrile as solvent B. The gradient was set: 0 to 2 min 5% B, from 2 to 10 min 5–20% B, from 10 to 15 min 20–30% B, from 15 to 20 min 30–35% B, from 20 to 60 min 35–80% B, from 60 to 65 min 80–85%, from 65 to 70 min 85–5% B. The flow rate was 0.89 mL/min, while the injector volume of samples was 20 μL. The used wavelength was 254 nm. The weight percentage was determined by Equation (3):

(3) $$\omega =\frac{c\left(component\right)}{c\left(extract\right)}\cdot 100\%$$

### 3.5. HPLC Method for Determination of Sugars and Their Derivatives

The other method was used to determine sugars and their derivatives from extracts used a Shimadzu Nexera HPLC system (Shimadzu, Kyoto, Japan) consisting of a DGU-20A SR degasser, LC-20AD XR pump, SIL-20AC XR autosampler, CTO-20AC column heater, RI and UV-PDA detector. The used column was a Rezex RHM-Monosaccharide H+ (300 × 7.8 mm, Phenomenex, Torrance, CA, USA). The temperature of column was 80 °C. The mobile phase was water with flow rate of 0.6 mL/min. UV detector was set at wavelength of 210 and 280 nm. The concentration of each component was calculated using standard calibration curves. The weight percentage of compounds was calculated by Equation (3).

Standard stock solutions for all analyzed bioactive compounds were prepared in a solvent and calibration was obtained at eight concentrations (concentration range 10.0, 20.0, 30.0, 50.0, 75.0, 100.0, 150.0, 200.0, mg/L). Linearity of the calibration curve was confirmed by *R*^2^ = 0.9997 for theobromine, *R*^2^ = 0.9997 for caffeine, *R*^2^ = 1 for epicatechin, *R*^2^ = 0.9998 for chlorogenic acid, *R*^2^ = 0.9997 for gallic acid, *R*^2^ = 0.9998 for glucose, *R*^2^ = 0.9997 for xylose, *R*^2^ = 0.9998 for 5-HMF, *R*^2^ = 0.9996 for levulinic acid, *R*^2^ = 0.9997 for lactic acid, *R*^2^ = 0.9997 for formic acid. LOD and LOQ are as follows: theobromine 0.182 mg/L, 0.552 mg/L; caffeine 0.145 mg/L, 0.439 mg/L; epicatechin 0.170 mg/L, 0.515 mg/L; chlorogenic acid 0.123 mg/L, 0.373 mg/L; gallic acid 0.146 mg/L, 0.442 mg/L; glucose 0.145 mg/L, 0.439 mg/L; xylose 0.0405 mg/L, 0.123 mg/L; 5-HMF 0.0125 mg/L, 0.0379 mg/L; levulinic acid 0.365 mg/L, 1.106 mg/L; lactic acid 0.285 mg/L, 0.864 mg/L; formic acid 0.428 mg/L, 1.297 mg/L.

### 3.6. Determination of DPPH Scavenging Activity

Antiradical activity of obtained extracts was determined using DPPH method described earlier [42]. The extracts were dissolved in ethyl acetate (250 μg/mL) and mixed with 0.3 mM DPPH radical solution. All measurements were done in triplicate. The absorbance was measured at 517 nm and DPPH scavenging activity was determined using Equation (4):

(4) $$\%\;DPPH\;activity=\frac{\left(A_{DPPH}+A_{b}\right)-A_{s}}{A_{DPPH}}*100$$

where are *A_DPPH_*—control absorbance, *A_b_*—sample absorbance, *A_s_*—sample absorbance mixed with DPPH solution.

### 3.7. Determination of Total Phenolic Content (TP)

Total phenolic (TP) content of the extracts was determined by a modified spectrophotometric method with Folin-Ciocalteu reagent, calibrated against gallic acid [43]. The results were calculated according to the calibration curves for gallic acid and TPs mass fraction, derived from triplicate analyses and expressed as mg of gallic acid equivalents (GAE) per g of the extracts. All these experiments were performed in triplicate.

### 3.8. Statistical Analysis

Experimental data were statistically analyzed using the commercial Design-Expert^®^ software (ver. 9, Stat-Ease Inc., Minneapolis, MN, USA). The analysis of variance (ANOVA) was also used to evaluate the quality of the fitted model. The test of statistical difference was based on the total error criteria with a confidence level of 95.0%. To understand the effect of independent variables on response variables, response plots were generated using the same software.

## 4. Conclusions

This is the first study that provides information on the application of SWE technology for the extraction of high-added value compounds from cocoa shells, a cocoa processing by-product. Subcritical water represents a good replacement for organic solvents and an environmentally friendly medium to treat different materials without the addition of catalyst. As a suitable raw material cocoa shell was selected, due to the fact that tonnes are disposed of as waste every year, hence the combination of this material and subcritical water to find optimal extraction parameters for obtaining important components seemed like a good idea. SWE was carried out in temperature range from 120 °C to 220 °C and reaction times from 15 min to 75 min using different solvent-solid ratios. The detected components were theobromine, theophylline, caffeine, catechin, epicatechin, gallic and chlorogenic acids, as well as some sugars and their derivatives. It was proved that theobromine concentration increases from 120 °C to 170 °C and after that it decreases. The explanation can be that detected caffeine converts into theobromine and at higher reaction parameters they both degrade into minor compounds. Also, as the temperature and reaction time increase, TP and antioxidant activity increase. Sugar concentrations mostly increase with increasing temperature and reaction time. The organic acids, as sugar degradation products, were obtained at higher temperature (220 °C).

In summary, use of an elevated temperature in SWE brings several advantages in terms of higher content of some valuable bioactive compounds, but at the higher temperatures, also more unwanted compounds, so called contaminants, were extracted, such as 5-HMF. Because of that it is important to optimize the extraction procedure. Cocoa shell, due to its bioactive components, has the potential to become a desirable raw material in a large spectrum of functional and pharmaceutical products.

## Figures and Tables

**Table 1:** Bioactive compounds detected in cocoa shell extracts according to BBD.

Run	Temperature (°C)	Time (min)	Solvent-Solid Ratio (mL/g)	Extract Yield, %	Theobromine % (*w*/*w*)	Caffeine % (*w*/*w*)	Theophylline % (*w*/*w*)	Gallic Acid % (*w*/*w*)	Epicatechin % (*w*/*w*)	Catechin % (*w*/*w*)	Chlorogenic Acid % (*w*/*w*)	TP (mg GAE/g Extract)	% DPPH Scavening
1	170	45	20	39.64	4.15	0.23	traces	traces	1.13	traces	traces	95.97	62.99
2	120	75	20	35.12	2.94	0.13	-	-	0.63	traces	traces	27.26	19.20
3	170	45	20	40.15	4.77	0.23	traces	traces	0.94	traces	traces	94.18	52.06
4	170	75	30	32.79	3.45	0.24	traces	-	0.58	traces	traces	57.01	48.36
5	120	45	30	25.39	1.63	0.07	-	-	0.59	traces	traces	43.67	31.27
6	170	75	10	32.54	4.32	0.19	traces	traces	0.44	traces	traces	91.10	64.48
7	120	45	10	53.16	1.31	0.04	-	-	0.41	traces	-	98.28	63.83
8	220	45	10	27.08	3.57	0.17	0.045	-	0.23	0.37	0.01	93.41	66.33
9	120	15	20	33.00	2.12	0.10	-	-	0.66	traces	-	94.44	65.46
10	170	15	10	28.89	3.83	0.29	-	traces	1.23	traces	traces	96.49	70.51
11	170	45	20	42.74	4.26	0.21	traces	traces	0.85	traces	traces	101.35	74.47
12	170	45	20	39.01	3.30	0.20	traces	traces	0.55	traces	traces	85.72	80.60
13	170	15	30	42.71	2.95	0.25	traces	-	3.29	traces	traces	33.41	20.24
14	170	45	20	39.72	3.57	0.24	traces	traces	0.47	traces	traces	113.41	71.89
15	220	45	30	33.42	3.27	0.16	0.04	-	0.19	0.29	0.01	117.51	83.62
16	220	15	20	35.76	3.27	0.14	0.07	-	0.38	0.07	0.01	44.95	33.67
17	220	75	20	30.10	3.65	0.18	0.17	-	0.25	0.45	0.03	130.33	91.69

**Table 2:** Regression coefficient of polynomial function of the most significant response surfaces.

Term	Coefficients	Standard Error	*F*-Value	*p*-Value
**Theobromine**				
Intercept	4.21	0.22		
*X*_1_	0.72	0.17	17.53	0.0041 *
*X*_2_	0.27	0.17	2.55	0.1540
*X*_3_	−0.22	0.17	1.60	0.2459
*X*_1_^2^	−1.20	0.24	25.84	0.0014 *
*X*_2_^2^	−0.011	0.24	0.002	0.9647
*X*_3_^2^	−0.56	0.24	5.59	0.0501
*X*_1_ *X*_2_	−1.20	0.24	0.20	0.6645
*X*_1_ *X*_3_	−0.011	0.24	0.40	0.5464
*X*_2_ *X*_3_	−0.56	0.24	0.000	0.9976
**Caffeine**				
Intercept	0.22	0.014		
*X*_1_	0.039	0.011	12.98	0.0087 *
*X*_2_	−0.0057	0.011	0.29	0.6085
*X*_3_	0.0036	0.011	0.12	0.7436
*X*_1_^2^	−0.11	0.015	51.39	0.0002 *
*X*_2_^2^	0.026	0.015	3.16	0.1185
*X*_3_^2^	−0.003	0.015	0.041	0.8450
*X*_1_ *X*_2_	0.000	0.015	0.000	1.0000
*X*_1_ *X*_3_	−0.0088	0.015	0.34	0.5800
*X*_2_ *X*_3_	0.023	0.015	2.40	0.1653
**Total phenols**				
Intercept	98.13	5.35		
*X*_1_	15.32	4.23	13.10	0.0085 *
*X*_2_	4.55	4.23	1.16	0.3178
*X*_3_	−15.96	4.23	14.22	0.0070 *
*X*_1_^2^	−2.58	5.83	0.20	0.6711
*X*_2_^2^	−21.30	5.83	13.33	0.0082
*X*_3_^2^	−7.33	5.83	1.58	0.2495
*X*_1_ *X*_2_	38.14	5.99	40.60	0.0004 *
*X*_1_ *X*_3_	19.68	5.99	10.81	0.0133 *
*X*_2_ *X*_3_	7.25	5.99	1.47	0.2653
**DPPH**				
Intercept	68.40	4.95		
*X*_1_	11.94	3.92	9.30	0.0186 *
*X*_2_	4.23	3.92	1.17	0.3156
*X*_3_	−10.21	3.92	6.80	0.0351 *
*X*_1_^2^	−2.76	5.40	0.26	0.6244
*X*_2_^2^	−13.13	5.40	5.91	0.0453 *
*X*_3_^2^	−4.37	5.40	0.66	0.4449
*X*_1_ *X*_2_	26.07	5.54	22.16	0.0022 *
*X*_1_ *X*_3_	12.46	5.54	5.06	0.0592
*X*_2_ *X*_3_	8.54	5.54	2.38	0.1671

*X*_1_: temperature; *X*_2_: time; *X*_3_: solvent-solid ratio; * *p* ≤ 0.05 significant.

**Table 3:** Analysis of variance (ANOVA) of the modelled responses.

Source	Sum of Squares	Degree of Freedom	Mean Square	*F*-Value	*p*-Value
**Theobromine**					
The recovery					
Model	13.06	9	1.45	6.14	0.0129
Residual	1.65	7	0.24		
Lack of fit	0.92	3	0.31	1.66	0.3117
Pure error	0.74	4	0.18		
Total	14.72	16			
*R*^2^ = 0.887					
**Caffeine**					
*The recovery*					
Model	0.064	9	0.007	7.75	0.0066
Residual	0.006	7	0.0009		
Lack of fit	0.005	3	0.002	9.24	0.0585
Pure error	0.0008	4	0.0002		
Total	0.071	16			
*R*^2^ = 0.909					
**Total phenols**					
The recovery					
Model	13,944.84	9	1549.43	10.81	0.0024
Residual	1003.19	7	143.31		
Lack of fit	584.97	3	194.99	1.86	0.2763
Pure error	418.22	4	104.55		
Total	14,948.03	16			
*R*^2^ = 0.933					
**DPPH**					
The recovery					
Model	6643.02	9	738.11	6.02	0.0137
Residual	858.97	7	122.71		
Lack of fit	364.94	3	121.65	0.98	0.4842
Pure error	494.03	4	123.51		
Total	7502.00	16			
*R*^2^ = 0.886					

**Table 4:** The concentrations of detected sugars and sugar degradation products in cocoa shell extract in % (*w*/*w*).

Run	Temperature (°C)	Time (min)	Solvent-Solid Ratio (mL/g)	Glucose	Rhamnose	Arabinose	Mannose	Xylose	Fucose	Levulinic Acid	Lactic Acid	Formic Acid	5-HMF	Furfural
1	170	45	20	0.89	1.82	5.85	-	2.93	1.70	-	-	-	Traces	Traces
2	120	75	20	-	-	-	1.17	-	-	-	-	-	-	-
3	170	45	20	2.76	1.71	3.27	-	3.61	1.46	-	-	-	Traces	Traces
4	170	75	30	3.21	2.84	4.57	-	3.94	2.04	-	-	-	Traces	Traces
5	120	45	30	-	-	-	0.91	-	-	-	-	-	-	-
6	170	75	10	2.84	2.35	3.92	-	3.66	1.88	-	-	-	Traces	Traces
7	120	45	10	-	-	-	0.68	-	-	-	-	-	-	-
8	220	45	10	2.67	5.74	5.71	-	5.18	5.20	10.95	6.69	1.96	Traces	Traces
9	120	15	20	-	-	-	0.27	-	-	-	-	-	-	-
10	170	15	10	1.02	1.09	1.13	-	2.97	1.05	-	-	-	Traces	Traces
11	170	45	20	1.19	1.53	3.48	-	3.56	1.32	-	-	-	Traces	Traces
12	170	45	20	1.34	-	3.78	-	3.25	1.23	-	-	-	Traces	Traces
13	170	15	30	1.32	1.47	2.88	-	3.22	0.98	-	-	-	Traces	Traces
14	170	45	20	1.53	1.94	3.09	-	2.90	1.66	-	-	-	Traces	Traces
15	220	45	30	4.61	5.93	6.48	-	4.22	4.70	11.83	7.07	2.37	0.35	0.69
16	220	15	20	2.36	4.20	4.59	-	4.60	4.26	6.37	6.38	0.36	0.96	2.62
17	220	75	20	4.22	6.00	6.57	-	5.84	5.65	7.44	4.18	3.25	0.030	0.41

## References

[1] Martin-Cabrejas M.A., Valiente C., Esteban R.M., Molla E. (1994). Cocoa hull: A potential source of dietary fibre. J. Sci. Food Agric..

[2] Nsor-Atindana J., Zhong F., Mothibe K.J. (2012). In vitro hypoglycemic and cholesterol lowering effects of dietary fiber prepared from cocoa (*Theobroma cacao* L.) shells. J. Funct. Food.

[3] Okiyama D.C.G., Navarro S.L., Rodrigues C.E.C. (2017). Cocoa shell and its compounds: Applications in the food industry. Trends Food Sci. Technol..

[4] EI-Saied H.M., Morsi M.K., Amer M.M.A. (1981). Composition of cocoa shell fat as related to cocoa butter. Z. Ernahrung..

[5] Redgwell R.J., Hansen C.E. (1999). Isolation and characterisation of cell wall polysaccharides from cocoa (*Theobroma cacao* L.) beans. Planta.

[6] Redgwell R., Trovato V., Merinat S., Curti D., Hediger S., Manez A. (2003). Dietary fibre in cocoa shell: Characterisation of component polysaccharides. Food Chem..

[7] Lecumberri E., Mateos R., Izquierdo-Pulido M., Ruperez P., Goya L., Bravo L. (2007). Dietary fibre composition, antioxidant capacity and physico-chemical properties of a fibre-rich product from cocoa (*Theobroma cacao* L.). Food Chem..

[8] Hartati I. (2010). Hydrotropic extraction of Theobromine from cocoa bean shell. Momentum.

[9] Hamzat R.A., Adeola O. (2011). Chemical evaluation of co-products of cocoa and kola as livestock feeding stuffs. J. Anim. Sci. Adv..

[10] Bruna C., Eichholz I., Rohn S., Kroh L.W., Huyskens-Keil S. (2009). Bioactive compounds and antioxidant activity of cocoa hulls (*Theobroma cacao* L.) from different origins. J. Appl. Bot. Food Qual..

[11] Nsor-Atindana J., Zhong F., Mothibe K.J., Bangoura M.L., Lagnika C. (2012). Quantification of total polyphenolic content and antimicrobial activity of cocoa (*Theobroma cacao* L.) bean shells. Pak. J. Nutr..

[12] Cvjetko Bubalo M., Vidović S., Radojčić Redovniković I., Jokić S. (2015). Green solvents for green technologies. J. Chem. Technol. Biot..

[13] Akiya N., Savage P.E. (2002). Roles of Water for Chemical Reactions in High-Temperature Water. Chem. Rev..

[14] Kubatova A., Miller D.J., Hawthorne B.S. (2001). Comparison of subcritical water and organic solvents for extracting kava lactones from kava root. J. Chromatogr. A.

[15] Scalbert A., Manach C., Morand C., Remesy C., Jimenez L. (2005). Dietary polyphenols and the prevention of diseases. Crit. Rev. Food Sci..

[16] Koffi E., Sea T., Dodehe Y., Soro S. (2010). Effect of solvent type on extraction of polyphenols from twenty three Ivorian plants. J. Anim. Plant Sci..

[17] Nazaruddin R., Seng L.K., Hassan O., Said M. (2006). Effect of pulp pre-conditioning on the content of polyphenols in cocoa beans (*Theobroma cacao*) during fermentation. Ind. Crop. Prod..

[18] Azizah A.H., Nik Ruslawati N.M., Swee Tee T. (1999). Extraction and characterization of antioxidant from cocoa by-products. Food Chem..

[19] Barbosa-Pereira L., Guglielmetti A., Zeppa G. (2018). Pulsed electric field assisted extraction of bioactive compounds from cocoa bean shell and coffee silverskin. Food Bioprocess Technol..

[20] Mazzutti S., Rodrigues L.G.G., Mezzomo N., Venturi V., Ferreira S.R.S. (2018). Integrated green-based processes using supercritical CO_2_ and pressurized ethanol applied to recover antioxidant compouds from cocoa (*Theobroma cacao*) bean hulls. J. Supercrit. Fluids.

[21] Ismail A., Lee C.Y. (2006). Antioxidative effects of extracts of cocoa shell, roselle seeds and a combination of both extracts on the susceptibility of cooked beef to lipid oxidation. J. Food Techol..

[22] Kim H., Keeney P.G. (1984). (-)-Epicatechin content in fermented and unfermented cocoa beans. J. Food Sci..

[23] Hammerstone J.F., Lazarus S.A., Mitchell A.E., Rucker R., Schmitz H.H. (1999). Identification of procyanidins in cocoa (*Theobroma cacao*) and chocolate using high-performance liquid chromatography/mass spectrometry. J. Agric. Food Chem..

[24] Hernández-Hernández C., Viera-Alcaide I., Sillero A.M.M., Fernández-Bolaños J., Rodríguez-Gutiérrez G. (2018). Bioactive compounds in Mexican genotypes of cocoa cotyledon and husk. Food Chem..

[25] Martínez R., Torres P., Meneses M.A., Figueroa J.G., Pérez-Álvarez J.A., Viuda-Martos M. (2012). Chemical, technological and in vitro antioxidant properties of cocoa (*Theobroma cacao* L.) co-products. Food Res. Int..

[26] Arlorio M., Coïsson J.D., Travaglia F., Varsaldi F., Miglio G., Lombardi G., Martelli A. (2005). Antioxidant and biological activity of phenolic pigments from *Theobroma cacao* hulls extracted with supercritical CO_2_. Food Res. Int..

[27] Maalik A., BukharI S.M., Zaidi A., Shah K.H., Khan F.A. (2016). Chlorogenic acid: A pharmacologically potent molecule. Acta Pol. Pharm..

[28] Santos L.F.S., Stolfo A., Salvador C.C.M. (2017). Catechin and epicatechin reduce mitochondrial dysfunction and oxidative stress induced by amiodarone in human lung fibroblasts. J. Arrhythm..

[29] Aprotosoaie A.C., Miron A., Trifan A., Luca V.S., Costache I.I. (2016). The cardiovascular effects of cocoa polyphenols—An overview. Diseases.

[30] Bispo M., Cristina M., Andrade J. (2002). Simultaneous determination of caffeine, theobromine, and theophylline by High-Performance Liquid Chromatography. J. Chromatogr. Sci..

[31] Timbie D.J., Sechrist L., Keeney P.G. (1978). Application of high-pressure liquid chromatography to the study of variables affecting theobromine and caffeine concentrations in cocoa beans. J. Food Sci..

[32] Carrillo L.C., Londoño-Londoño J., Gil A. (2014). Comparison of polyphenol, methylxanthines and antioxidant activity in Theobroma cacao beans from different cocoa-growing areas in Colombia. Food Res. Int..

[33] Gummadi S.N., Bhavya B., Ashok N. (2012). Physiology, biochemistry and possible applications of microbial caffeine degradation. Appl. Microbiol. Biotechnol..

[34] Sunil Paul M.M., Aravind U.K., Pramod G., Saha A., Aravindakumar C.T. (2014). Hydroxyl radical induced oxidation of theophylline in water: A kinetic and mechanistic study. Org. Biomol. Chem..

[35] Ravber M., Knez Ž., Škerget M. (2015). Simultaneous extraction of oil- and watersoluble phase from sunflower seeds with subcritical water. Food Chem..

[36] Plaza M., Turner C. (2015). Pressurized hot water extraction of bioactives. Trend. Anal. Chem..

[37] Hess E.H. (1968). Process for Manufacturing Flavouring Material from Cocoa Shell-Containing Chocolate Manufacturing by Products. U.S. Patent.

[38] Kleinert J. (1982). Process for Producing Dietary Fibre for Improving the Digestive Properties of Foods and Drinks. EP Application Patent.

[39] Déniela M., Haarlemmer G., Roubaud A., Weiss-Hortala E., Fages J. (2016). Energy valorisation of food processing residues and model compounds by hydrothermal liquefaction. Renew. Sustain. Energy Rev..

[40] Déniela M., Haarlemmer G., Roubaud A., Weiss-Hortala E., Fages J. (2017). Hydrothermal liquefaction of blackcurrant pomace and model molecules: Understanding of reaction mechanisms. Sustain. Energy Fuels.

[41] Bas D., Boyacı I.H. (2007). Modeling and optimization I: Usability of response surface methodology. J. Food Eng..

[42] Jokić S., Bijuk M., Aladić K., Bilić M., Molnar M. (2016). Optimization of supercritical CO_2_ extraction of grape seed oil using response surface methodology. Int. J. Food Sci. Technol..

[43] Jakobek L., Šeruga M., Novak I., Medvidović-Kosanović M. (2007). Flavonols, phenolic acids and antioxidant activity of some red fruits. Deut. Lebensm-Rundsch..

